# The Sex Determination Gene *transformer* Regulates Male-Female Differences in *Drosophila* Body Size

**DOI:** 10.1371/journal.pgen.1005683

**Published:** 2015-12-28

**Authors:** Elizabeth J. Rideout, Marcus S. Narsaiya, Savraj S. Grewal

**Affiliations:** 1 Department of Biochemistry and Molecular Biology, University of Calgary, Calgary, Alberta, Canada; 2 Department of Cellular and Physiological Sciences, University of British Columbia, Vancouver, British Columbia, Canada; University of California Davis, UNITED STATES

## Abstract

Almost all animals show sex differences in body size. For example, in *Drosophila*, females are larger than males. Although *Drosophila* is widely used as a model to study growth, the mechanisms underlying this male-female difference in size remain unclear. Here, we describe a novel role for the sex determination gene *transformer* (*tra*) in promoting female body growth. Normally, Tra is expressed only in females. We find that loss of Tra in female larvae decreases body size, while ectopic Tra expression in males increases body size. Although we find that Tra exerts autonomous effects on cell size, we also discovered that Tra expression in the fat body augments female body size in a non cell-autonomous manner. These effects of Tra do not require its only known targets *doublesex* and *fruitless*. Instead, Tra expression in the female fat body promotes growth by stimulating the secretion of insulin-like peptides from insulin producing cells in the brain. Our data suggest a model of sex-specific growth in which body size is regulated by a previously unrecognized branch of the sex determination pathway, and identify Tra as a novel link between sex and the conserved insulin signaling pathway.

## Author Summary

Female-biased sexual size dimorphism is common in invertebrates, yet the mechanisms underlying increased female body size remain unclear. We uncovered a key role for sex determination gene *transformer* (*tra*) in promoting increased growth in females. Interestingly, we found that sex differences in body size are regulated by Tra in a pathway that is separate of the canonical sex determination pathway, and of other aspects of sexual dimorphism. Instead, Tra function in the fat body regulates growth in a non cell-autonomous manner by regulating the secretion of insulin-like peptides from the brain. This novel Tra-insulin link we describe may have implications for other sexually dimorphic phenotypes in *Drosophila* (*eg*. lifespan, stress resistance), many of which are also regulated by insulin.

## Introduction

*Drosophila* is a well-established model to study the mechanisms that control animal growth [1, 2]. *Drosophila* body size is determined by various developmental cues that coordinate tissue patterning with growth, and by environmental cues such as nutrients and oxygen, that regulate whole body metabolism. One important, but often overlooked, determinant of size in *Drosophila* is sex–adult females are significantly, and visibly, larger than males [3, 4]. This sexual size dimorphism (SSD) arises due to differences in larval growth: males and females have a similar overall duration of larval development, but females achieve critical weight at a larger size and grow more during the terminal growth period [5]. While over two decades of genetic research have identified many conserved signaling pathways that link developmental and environmental cues to the control of tissue and body size [6–9], the genetic and physiological mechanisms that account for the larger female body size remain unclear.

In flies, sex is determined by the ratio of sex chromosomes to autosomes (X:A) [10]. In females, the X:A ratio is 1, and a functional protein is produced from the *Sex-lethal* (*Sxl*) locus [11, 12]. In males, the X:A ratio is 0.5, and no Sxl is produced. Sxl is a master regulator of female sexual development (*eg*. sexual differentiation, reproduction), and Sxl mutant females are smaller than wild-type females. This is due, in large part, to the sex-specific splicing of its downstream target gene *transformer* (*tra*) [13–16]. As a result of this Sxl-dependent splicing, a functional Tra protein is produced in females, but not males. Tra is a splicing factor, and has only two known direct targets: *doublesex* (*dsx*) and *fruitless* (*fru*) [17–22]. While Tra is thought to mediate most of Sxl’s effects on sex determination, the control of sex differences in body size is thought to be independent of the Tra/Dsx/Fru branch of the sex determination pathway [23]. Here, we identify for the first time a role for Tra as a key regulator of SSD in *Drosophila*. Further, we show that Tra’s effects on SSD are mediated by a novel pathway that is independent of *dsx* and *fru*, and of other aspects of sexual dimorphism.

## Results

### Tra regulates sex differences in body size

**Fig 1:**
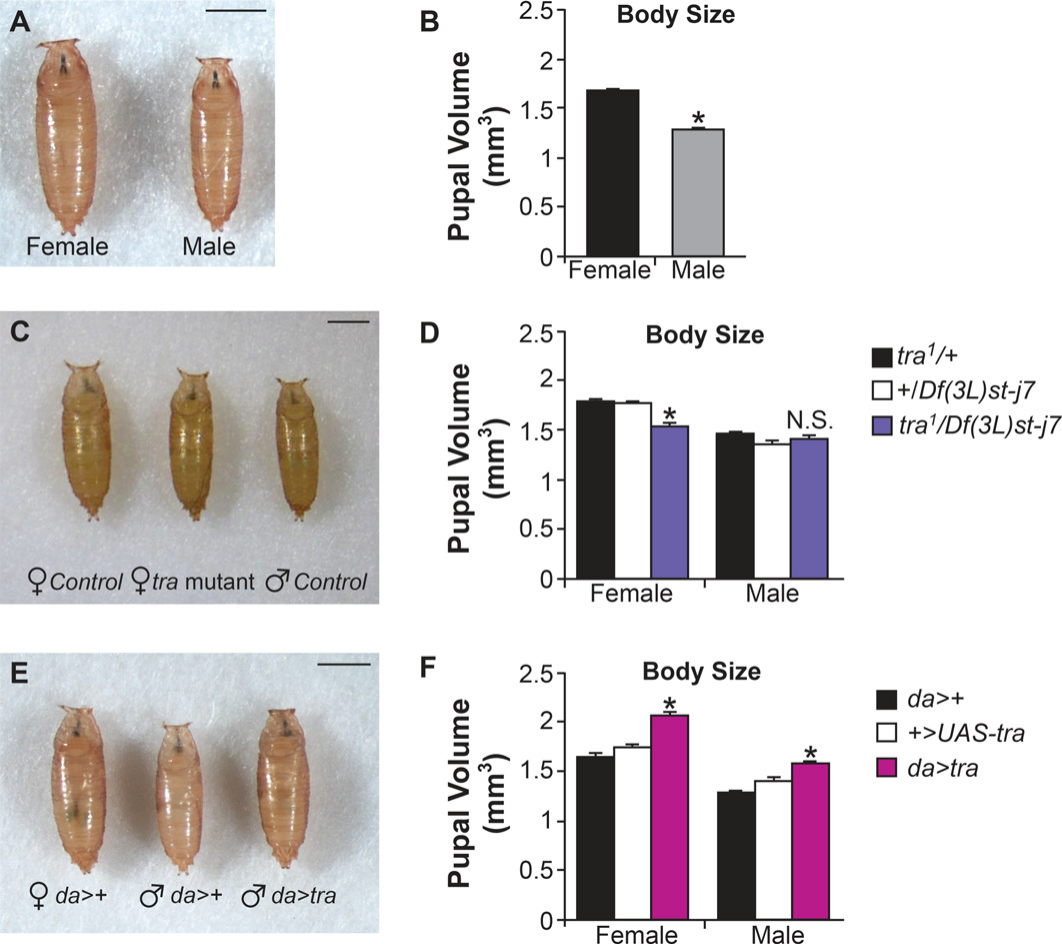
Sex determination gene *transformer* regulates sex differences in body size. (A) Photograph of *w* ^*1118*^ male and female pupae, approximately 48 hr after puparium formation. (B) Quantification of pupal volume in *w* ^*1118*^ male and female pupae (n>60). Females are approximately 30% larger in size than males (p = 1.8 x 10^−43^, Student’s t-test). (C) Photograph of control male and female pupae (*tra* ^*1*^ */+*) compared to a *tra* mutant female (*tra* ^*1*^/*Df(3L)st-j7*). (D) Pupal volume in *tra* mutant females is significantly reduced compared to control females (p = 7.1 x 10^−7^;2.3 x 10^−6^, one-way ANOVA followed by Tukey HSD post-hoc test). Male pupal volume is unaffected (p = 0.91;0.87, one-way ANOVA followed by Tukey HSD post-hoc test). n>60 for all genotypes. (E) Photograph of male and female control pupae (*da>+*) compared to males with ubiquitous overexpression of Tra (*da>tra*). (F) *da>tra* males and females are significantly larger than control animals (p = 0;0 and 0;3.6 x 10^−5^, one-way ANOVA followed by Tukey HSD post-hoc test). (A, C, E) Scale bars = 1 mm. * indicates a significant difference from control genotypes. N.S. means not significantly different from both control genotypes. The p-values indicated are listed in the following order: difference between the GAL4/UAS genotype and the GAL4 control (or first genotype control);difference between the GAL4/UAS genotype and the UAS control (or second genotype control). A list of all p-values obtained from the Tukey HSD post-hoc test is provided in S1 Table.

Female and male *Drosophila* larvae show no difference in their rate of development [5]. However, by the end of larval life, female body size is approximately 30% larger than male body size (Fig 1A and 1B). These differences are not due to sex differences in food intake or feeding behaviour (S1A and S1B Fig). Although the prevailing view is that *tra* does not regulate sex differences in body size [24], one study showed that adult weight in *tra* mutant females was reduced compared to wild-type females [25]. However, this weight reduction can be explained by the lack of ovaries in *tra* mutant females. We therefore tested whether the decreased weight was due to an effect of *tra* on growth by measuring pupal volume in *tra* mutant animals. We found that body size was significantly reduced in *tra* mutant females compared to wild-type females (Fig 1C and 1D). Thus while wild-type females are 30% larger in body size than males, *tra* mutant females are only 10% larger than males. This suggests that *tra* contributes to establishing SSD in *Drosophila*. Body size was unchanged in *tra* mutant males, consistent with the lack of a functional Tra protein in males (Fig 1D). We also performed loss-of-function experiments with *tra*, using an RNAi transgene directed against *tra’s* splicing co-factor *tra2* (*UAS-tra2-RNAi*). We found that ubiquitous expression of the *UAS-tra2-RNAi* transgene using the *Act5c-GAL4* driver successfully transformed female animals into phenotypic males (S1C Fig), and led to a reduction in body size (S1D Fig).

We next examined whether lack of Tra expression in males could explain their smaller body size. Ubiquitous expression of a *UAS-tra* transgene using the *daughterless* (*da*)-GAL4 driver led to a significant increase in body size in males (Fig 1E and 1F). Interestingly, overexpression of Tra also stimulated growth in females, showing Tra has growth-promoting effects in both sexes. While previous studies have shown that high levels of Tra expression can cause artifacts such as lethality [26], this is the first report of an alternative splicing factor promoting body growth in *Drosophila*. To further confirm these Tra-dependent changes in body size, we measured adult weight. In order to ensure that *tra’s* effects on adult weight are not confounded by its effects on ovary or testis development, we weighed 5-day-old animals from which the gonads were removed by dissection. As with pupal volume, we found that *tra* mutant females, but not *tra* mutant males, were significantly smaller than controls (S2A Fig). Overexpression of *UAS-tra* caused an increase in body weight in males (S2B Fig). Together, these results suggest that male-female differences in body size are created in part by the presence of Tra in females, and the absence of Tra in males. This defines a new role for Tra in the regulation of sex differences in body growth.

### Tra regulates growth in a cell-autonomous manner

**Fig 2:**
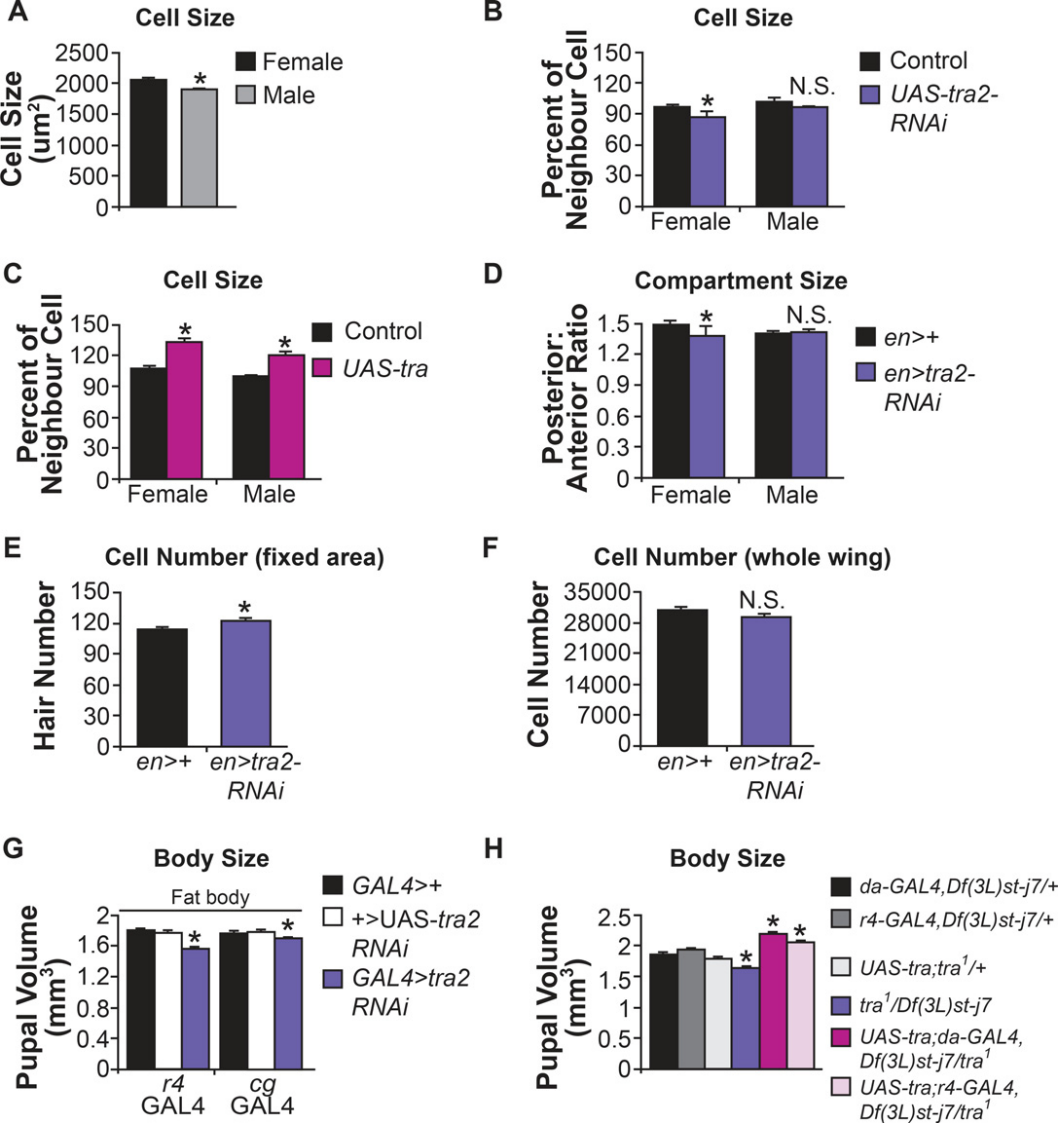
*tra* regulates growth in a cell-autonomous, and a non cell-autonomous manner. (A) Fat body cell size is significantly larger in wild-type females (p = 7.6 x 10^−7^; Student’s t-test). (B) Mosaic expression of a *tra2-RNAi* transgene in the fat body causes a significant reduction in cell size in females (p = 0.024; Student’s t-test). Male cell size is unaffected by expression of the *tra2-RNAi* transgene (p = 0.063; Student’s t-test). (C) Mosaic overexpression of Tra in the fat body stimulates cell growth to increase cell size in both females and males (p = 6.8 x 10^−10^ and 5.6 x 10^−9^, respectively; Student’s t-test). (D) Expression of a *tra2-RNAi* transgene in the posterior compartment of the wing using *en-GAL4* caused a significant reduction in the ratio of posterior:anterior (p = 1.5 x 10^−6^; Student’s t-test). Male posterior:anterior ratio was unaffected (p = 0.062; Student’s t-test). (E) The number of wing hairs in a fixed area in females expressing the *tra2-RNAi* transgene in the posterior compartment of the wing is greater than in control females (p = 0.0034; Student’s t-test). (F) The total number of cells in the posterior compartment of the wing is not significantly different in females upon expression of the *tra2-RNAi* transgene compared to wild-type females (p = 0.063; Student’s t-test). (G) Tissue-specific expression of the *tra2-RNAi* transgene in the fat body significantly decreased pupal volume (p = 0;0.0006 (r4), 0.008;0.001 (cg), one-way ANOVA followed by Tukey HSD post-hoc test). (H) Ubiquitous, or fat body-specific, overexpression of *UAS-tra* rescues the decreased body size of *tra* mutant females (one-way ANOVA followed by Tukey HSD post-hoc test, see S1 Table for full set of statistical comparisons between genotypes). * indicates a significant difference from control genotypes. N.S. means not significantly different from both control genotypes. The p-values indicated are listed in the following order: difference between the GAL4/UAS genotype and the GAL4 control;difference between the GAL4/UAS genotype and the UAS control.

As with body size, female cell size in the wing [27], and the fat body (Fig 2A) are larger. Since one previous study showed that wing cell size in *tra* mutant females was intermediate in size between female and male cells [25], we tested whether Tra expression could mediate cell-autonomous effects on growth by expressing either *UAS-tra* or the *UAS-tra2-RNAi* transgenes in the fat body (polyploid cells) and the wing disc cells (mitotic cells) of developing larvae. We found that *flp*-out-mediated mosaic expression of *UAS-tra2-RNAi* in female fat body caused a significant reduction in cell size (Fig 2B). Consistent with the lack of a functional Tra protein in males, similar *UAS-tra2-RNAi* expression in males did not affect cell size (Fig 2B). In contrast, overexpression of *UAS-tra* in fat body cells was sufficient to increase cell size in both sexes (Fig 2C). We next examined whether Tra expression could affect sex differences in another larval tissue, the wing disc. Using *engrailed* (*en*)-*GAL4*, we expressed either *UAS-tra2-RNAi* or *UAS-tra* transgenes in the posterior compartment of the wing, and measured compartment size. We found a significant reduction in compartment size in female wings when we knocked down Tra function by *UAS-tra2-RNAi* expression (Fig 2D). This effect was also seen when we used a second *UAS-tra2-RNAi* transgene (S3A Fig). In contrast, male compartment size was unaffected (Fig 2D). To determine whether this reduction in compartment size was due to a decrease in cell number or cell size, we counted wing hairs in a fixed area in the posterior compartment in females. Each wing cell secretes one hair, thus by counting wing hairs we can accurately determine how many cells are present in a specific area. We found that the number of cells in the counting area was significantly increased in the compartments expressing *UAS-tra2-RNAi* compared to controls (Fig 2E). This suggests that the reduction in compartment size is due to a reduction in cell size, rather than cell number. Indeed, the estimated cell number in the posterior compartment of the wing was not significantly altered by expression of *UAS-tra2-RNAi* (Fig 2F). Overexpression of *UAS-tra* using *en-GAL4* caused no significant increase in compartment size in either males or females (S3B Fig). Together, these results demonstrate a cell-autonomous requirement for Tra in females to promote increased female cell size in both mitotic and endoreplicating cells. This result supports previous findings from early gynandromorph studies, where sex differences in cell size were regulated in a cell-autonomous manner [28]. More recently, Sxl expression in the wing disc was shown to promote growth [29]; however, altering either the X:A ratio or Sxl expression also affects the process of dosage compensation. Since Tra does not affect this process [30], our results demonstrate that sex differences in cell size can be uncoupled from dosage compensation.

### Tra function in the fat body controls growth in a non cell-autonomous manner

An emerging literature in *Drosophila* has highlighted the importance of noncell-autonomous signaling in the control of body growth [31–34]. This signaling relies on organ-to-organ endocrine communication and is particularly important in controlling body growth in response to dietary nutrients. We therefore tested whether sex differences in overall body size may also involve non-autonomous effects of Tra function specific tissues. We used a number of tissue-specific GAL4 drivers to express the *UAS-tra2-RNAi* transgene in larvae, and measured pupal volume in these animals. We found that loss of Tra in the fat body caused a significant reduction in female body size (Fig 2G). This reduction in body size was also observed in females expressing a second *UAS-tra2-RNAi* transgene in the fat body (S3C Fig). Expression of *UAS-tra2-RNAi* in neurons, glia, ring gland or in muscle did not reproduce the female-specific effects of the fat body (S4A Fig), and male body size was unaffected by expression of the *UAS-tra2-RNAi* transgene in any tissue (S4B Fig). We next asked whether expression of *UAS-tra* in specific tissues was sufficient to drive an increase in body size. Tissue-specific expression of *UAS-tra* using a panel of GAL4 drivers did not significantly affect body size in wild-type females or males (S5A and S5B Fig). We then asked whether tissue-specific expression of Tra could rescue the body size defects of *tra* mutant females. We found that ubiquitous, or fat-specific, expression of Tra was sufficient to rescue a normal body size to *tra* mutant females (Fig 2H). Together, these results suggest that the decreased body size in *tra* mutant females is due to fat-specific loss of Tra function. Tra controls many aspects of sexual differentiation, including gonad and germline differentiation, and previous studies in *C*. *elegans* showed that the germline can influence body growth [35]. We therefore tested whether Tra expression in these tissues could explain its effects on female body size. However, we found that gonad- or germline-specific expression of a *UAS-tra2-RNAi* transgene using the *c587-GAL4* or *nanos* (*nos*)-*GAL4* drivers, respectively, caused no significant reduction in body size in females (S6A and S6B Fig). Similarly, body size was unaffected in males and females completely lacking a germline (the progeny of *tudor* ^*1*^ homozygous mutant females crossed to wild-type males; S6C Fig). Decreased body size in *tra* mutant females is therefore not due to the presence of a male gonad or germline. Instead, our results suggest the sex of the fat body, as determined by Tra expression, controls body growth in a non cell-autonomous manner.

### Tra’s regulation of body size is independent of *fru* and *dsx*

**Fig 3:**
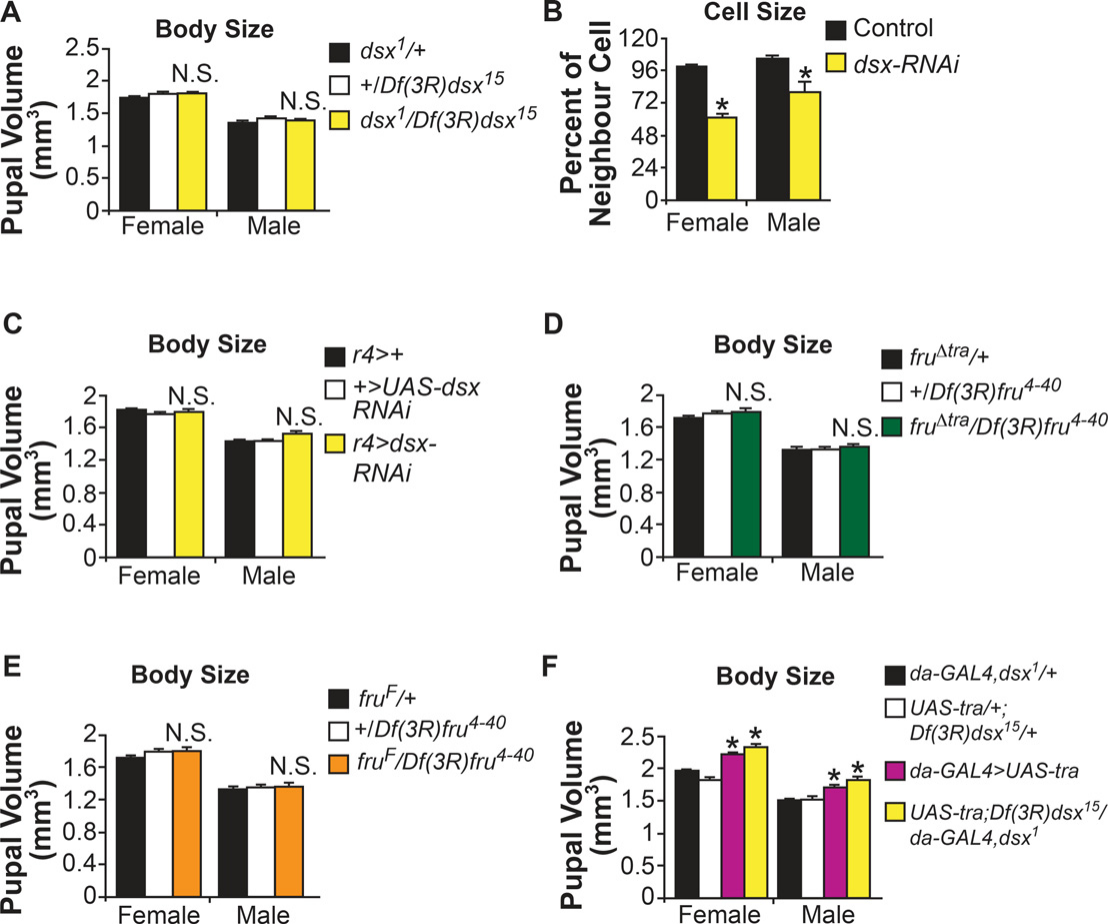
Sex determination genes *fru* and *dsx* do not affect body size. (A) Pupal volume in *dsx* mutant males and females (*dsx* ^*1*^ */Df(3R)dsx* ^*15*^) is not significantly different from control males and females (p = 0.92;1 and 0.99;0.99, respectively, one-way ANOVA followed by Tukey HSD post-hoc test). (B) Mosaic expression of *dsx-RNAi* in fat cells causes a significant reduction in cell size in both females (p = 1.1 x 10^−24^, Student’s t-test) and males (p = 2.8 x 10^−10^, Student’s t-test). (C) Expression of *dsx-RNAi* in the fat body does not alter body size in females or males (p = 0.95;0.97 and 0.24;0.28, one-way ANOVA followed by Tukey HSD post-hoc test). (D) Females lacking the Tra-binding site in *fru* P1 transcripts (*fru* ^*Δtra*^) ectopically express Fru^M^ in the central nervous system (CNS). Pupal volume in *fru* ^*Δtra*^ */Df(3R)fru* ^*4-40*^ females was not significantly different than in control females (p = 0.46;0.99, one-way ANOVA followed by Tukey HSD post-hoc test). Male body size was also unaffected (p = 0.99;0.99, one-way ANOVA followed by Tukey HSD post-hoc test). (E) Males with the *fru* ^*F*^ allele of *fru* have constitutively female-specific splicing of *fru* P1 transcripts, and thus produce no Fru^M^ in the CNS. Pupal volume in *fru* ^*F*^ */Df(3R)fru* ^*4-40*^ males was not significantly different than control males (p = 1;0.63, one-way ANOVA followed by Tukey HSD post-hoc test). Female pupal volume was unaffected (p = 1;0.99, one-way ANOVA followed by Tukey HSD post-hoc test). (F) Ubiquitous overexpression of Tra increases body size in males and females even in a *dsx* mutant background. Thus, no significant difference in body size is present between females overexpressing Tra in a wild-type, or *dsx* mutant background (p = 0.436, one-way ANOVA followed by Tukey HSD post-hoc test). Similar results were observed in males (p = 0.39, one-way ANOVA followed by Tukey HSD post-hoc test). * indicates a significant difference from control genotypes. N.S. means not significantly different from both control genotypes. The p-values indicated are listed in the following order: difference between the GAL4/UAS genotype and the GAL4 control;difference between the GAL4/UAS genotype and the UAS control. A list of all p-values obtained from the Tukey HSD post-hoc test is provided in S1 Table.

Tra is a splicing factor, and has only two known direct targets: *doublesex* (*dsx*) and *fruitless* (*fru*) [17–22]. Dsx is expressed in a handful of tissues throughout the body and in a restricted expression pattern in the central nervous system (CNS) in both males and females [36–40]. In females, Tra binding to *dsx* pre-mRNA causes a female-specific Dsx isoform to be produced (Dsx^F^). In males, which express no functional Tra protein, a default splice in *dsx* pre-mRNA generates a male-specific isoform of Dsx (Dsx^M^) [19, 20]. Tra binding to the pre-mRNA of transcripts from the *fru* P1 promoter causes the introduction of a stop codon, and no Fru P1 protein is expressed in females. In males, the lack of Tra leads to the use of a default splice in *fru* P1 transcripts, generating a male-specific Fru P1 protein (Fru^M^) [17, 18]. Fru^M^ expression is limited to males in approximately 2000 neurons in the CNS and peripheral nervous system (PNS) [18, 41]. Importantly, *dsx* and *fru* are thought to mediate most, if not all, effects of Tra on sex determination and behaviour [42, 43]. We therefore tested whether either gene was required for Tra’s effects on growth. We first examined whether mutants lacking Dsx or Fru^M^ expression phenocopied any of Tra’s effects on growth. We found that *dsx* mutant animals (genotype *dsx* ^*1*^ */Df(3R)dsx* ^*15*^), had no significant difference in pupal volume compared to controls in either males or females (Fig 3A). We also examined the effect of Dsx knockdown in the fat body using a *UAS-dsx RNAi* line. Using the *flp-*out system, we found that mosaic expression of *UAS-dsx RNAi* led to reduced fat cell size in both males and females, suggesting that *dsx* regulates cell size in this tissue (Fig 3B). However, expression of *UAS-dsx-RNAi* throughout the fat body using *r4-GAL4* did not recapitulate the non cell-autonomous reduction of body size observed upon Tra inhibition in this tissue (Fig 3C). While it seems counterintuitive that Dsx causes a reduction in size in the fat body, but does not affect body size, *dsx* expression is restricted to specific tissues in larvae [37, 39]. Thus while loss of *dsx* may affect cell size in a relatively small number of tissues, the expression may not be broad enough to cause a reduction in overall body size. Also, in the context of our findings with *tra*, loss of *dsx* throughout the fat body does not phenocopy the non cell-autonomous effects of loss of Tra on body size. Similar to *dsx*, we found that males lacking Fru^M^ expression, or females ectopically expressing Fru^M^ proteins [44], showed no difference in body size compared to controls (Fig 3D and 3E). We next asked whether *dsx* was required for Tra-induced growth. Using *da-GAL4* to overexpress Tra in a *dsx* mutant background, we found that Tra’s ability to drive body growth was unaffected by loss of *dsx* (Fig 3F). Our results suggest Tra controls growth in a pathway that is independent of its effects on sexual differentiation and behaviour.

### IIS, and not TOR, is required for sex differences in body growth

**Fig 4:**
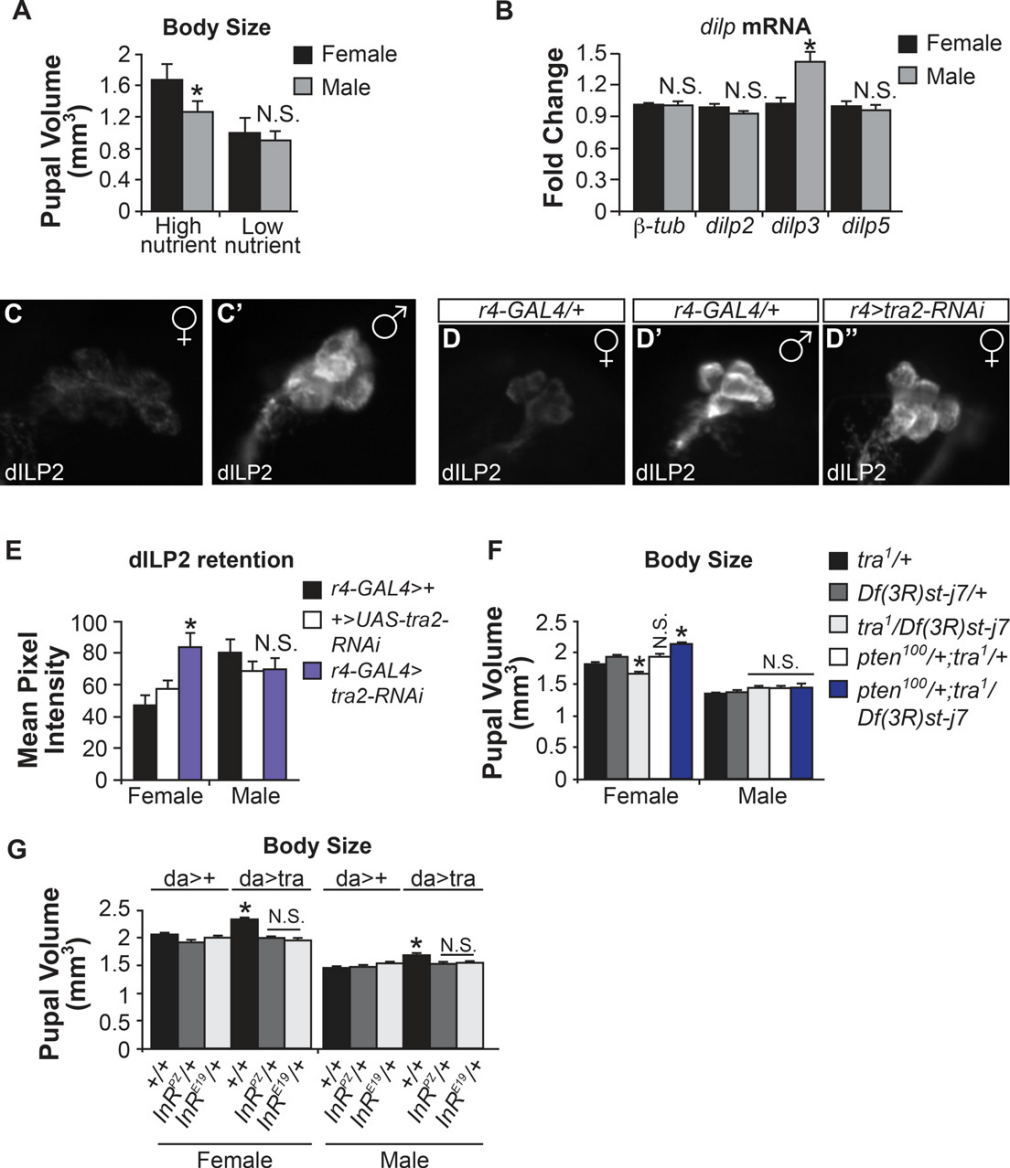
*tra* function in the fat body regulates male-female differences in dILP2 secretion. (A) Females are normally 30% larger than males in nutrient-rich (high IIS/TOR activity); however, in nutrient-poor food, sex differences in body size are abolished (p = 0.082, one-way ANOVA followed by Tukey HSD post-hoc test). (B) Male and female transcript levels of *insulin-like peptides 2*, *3 and 5* (*dilp2*, *dilp3*, *dilp5*) were analyzed in larval carcasses devoid of fat body at 120 hr AEL at 25°C. Levels of *dilp2* and *dilp5* were not different between males and females (p = 0.051, 0.19, Student’s t-test), but levels of *dilp3* were significantly higher in males (p = 0.0004; Student’s t-test). (C) Males (C’) have significantly higher dILP2 retention in the insulin producing cells (IPCs) in the brain than females (C) (p = 0.001). (D-D”,E) Levels of dILP2 retention in females expressing a *tra2-RNAi* transgene specifically in the fat body (*r4-GAL4*) are increased compared to control females (p = 0.001;0.025, Student’s t-test). (F) Loss of *tra* significantly reduces body size in females. However, body size in *tra* mutant animals that also have loss of one copy of PTEN, a repressor of IIS, rescues the reduced size of *tra* mutant females, but has no effect on *tra* mutant males (see S1 Table for complete list of p-values from one-way ANOVA followed by Tukey HSD post-hoc test). (G) Ubiquitous expression of *UAS-tra* significantly increases body size in males and females (p = 0;0 and 0;0, one-way ANOVA followed by Tukey HSD post-hoc test). However, in animals heterozygous for loss of one copy of the *insulin receptor* gene (*InR* ^*PZ*^, *InR* ^*E19*^), the increased growth driven by *UAS-tra* overexpression is blocked (see S1 Table for complete list of p-values from one-way ANOVA followed by Tukey HSD post-hoc test). * indicates a significant difference from control genotypes. N.S. means not significantly different from both control genotypes. The p-values indicated are listed in the following order: difference between the GAL4/UAS genotype and the GAL4 control;difference between the GAL4/UAS genotype and the UAS control.

In *Drosophila*, the conserved insulin/insulin-like growth factor signaling (IIS) and Target-of-Rapamycin (TOR) pathways are two main regulators of tissue and body growth [7, 45, 46]. Both pathways play a central role in linking dietary nutrients to regulation of larval metabolism and growth [34, 47–49]. We therefore tested whether IIS/TOR also plays a role in creating sex differences in body size. We first measured pupal volume in males and females grown in either nutrient-rich food (which promotes high levels of IIS/TOR signaling), or in food with reduced nutrition (which inhibits IIS/TOR signaling). We found that sex differences in body size were abolished in low nutrient conditions (Fig 4A). Since previous studies have shown that IIS/TOR can act in separate pathways to activate downstream effectors [48, 50], we wanted to specifically inhibit the TOR pathway, and examine the effects on body size. When we grew larvae on food containing rapamycin, a specific TOR inhibitor, we found an overall reduction in body size in both sexes; however, the SSD between males and female remained at 25% (S7A Fig). Thus IIS, but not TOR, is required for male-female body size differences. This finding is consistent with a recent study that showed sex differences in adult body weight were eliminated in animals heterozygous for two hypomorphic mutations in the *insulin receptor* (*InR*) gene [5].

We next examined whether we could detect any sex differences in IIS activity during development. The serine/threonine kinase Akt is phosphorylated and activated downstream of IIS. Measuring the ratio of the phosphorylated active form of Akt (P-Akt) to total Akt therefore provides a read-out of IIS activity. When we compared male and female larvae collected 96 hr and 120 hr after laying (AEL) at 25°C, we found that females had a significantly higher ratio of P-Akt:Akt at 120 hr AEL (S7B and S7C Fig). To further confirm higher IIS activity in females during development, we used an antibody staining in the larval fat body to detect the subcellular localization of the transcription factor FOXO. When IIS activity is high, FOXO is phosphorylated by P-Akt, and is evenly distributed throughout the cytoplasm and nucleus. When IIS is inhibited, FOXO re-localizes to the nucleus, where it regulates expression of its target genes [51]. We found the nuclear:cytoplasmic ratio of FOXO was significantly higher in males than in females, further suggesting that males have lower levels of IIS activity (S7D and S7E Fig). We found no significant male-female differences in mRNA levels of previously described *foxo* targets such as *InR*, *4E-BP*, or *dilp6* (S7F Fig) [52–54]. This may be due to additional FOXO-independent factors required for their expression [55,56]. While the differences in IIS we report here are not as dramatic as seen with genetic or starvation-mediated perturbation of IIS, they are consistent with females having a modest increase in IIS activity compared to males.

### Tra function in the fat body affects ILP release

To understand how females achieve a modest increase in IIS compared to males, we examined insulin-like peptide (ILP) expression in larval insulin-producing cells (IPCs). The IPCs express three *Drosophila* ILPs (*dilps 2*,*3* and *5*) [57, 58]. Nutrients have been shown to regulate both mRNA transcription and secretion of these *dilps* [33, 57, 58]. Thus in response to amino acid input to the fat body, an as-yet-unidentified secreted factor is released that acts upon the IPCs in the brain to trigger dILP2 and dILP5 release into the larval hemolymph. These dILPs bind to the insulin receptor on target cells to activate IIS and promote body growth. In contrast, when nutrient abundance is low, the fat-to-brain signal is reduced and secretion of dILPs is inhibited, leading to decreased systemic IIS and body growth. Given our finding that Tra function in the fat body is required for normal growth in females, we examined whether Tra expression in the fat body influences brain dILPs.

We first examined dILP transcript levels and release in wild-type males and females. Using qRT-PCR, we found that only *dilp3* transcript levels were different between the sexes, where males had a significant increase in *dilp3* compared to females (Fig 4B). We next wanted to determine whether we could detect any differences in dILP secretion. This can be assayed by immunostaining for dILP2 expression in the IPCs. When dILP secretion is high, dILP2 levels seen in IPC are low. Conversely, when secretion is decreased, dILP2 levels in the IPCs are higher [33]. When we compared males and females, we found that male IPCs had a significantly higher average pixel intensity with anti-dILP2 (Fig 4C). Since *dilp2* transcript levels are not different between males and females (Fig 4B), this result suggests that dILP2 secretion is higher in females than males.

Given the importance of the fat body as a regulator of IPC dILP release, we tested whether male-female differences in dILP2 secretion occur as a result of Tra expression in the female fat body. Using *r4-GAL4*, we expressed the *UAS-tra2-RNAi* transgene to inhibit Tra function specifically in the fat body, and measured *dilp* transcript levels (in the larval carcass that was devoid of fat body), or ILP2 staining intensity in the IPCs. We found that loss of Tra in the fat body did not affect transcript levels of *dilp2*, *dilp3* or *dilp5* in either males or females (S7G Fig). However, the average pixel intensity of dILP2 staining in the IPCs was significantly higher in *r4>tra2-RNAi* females compared to control females (Fig 4D and 4E). Male dILP2 levels were unchanged by loss of Tra function in the fat (Fig 4E). These results suggest that Tra expression in the female fat body can enhance levels of dILP secretion compared to males, to control systemic insulin signaling and consequently body size.

To test this, we measured pupal volume in *tra* mutants in which we genetically increase IIS activity via heterozygous loss of PTEN, a known inhibitor of IIS. We found that loss of one copy of PTEN rescued the decreased body size in *tra* mutant females (Fig 4F). We next wanted to test whether a reduction in IIS could suppress the ability of Tra to drive growth. Using *da-GAL4* to drive ubiquitous expression of the *UAS-tra* transgene, we measured body size in larvae heterozygous for null or hypomorphic alleles of the InR. This genetic inhibition of IIS blocked Tra-induced overgrowth (Fig 4G). Together, these results support a model of sex-specific growth in which Tra function in the female fat body stimulates the release of dILPs from the IPC. Higher dILP levels stimulate IIS activity to promote increased body growth in females. Overall, our results identify sex determination gene Tra as an additional regulator of the highly conserved IIS pathway.

## Discussion

In almost all animals, sex is an important determinant of body size [4]. While sex hormones have been shown to control the rate and duration of growth in mammals to achieve SSD, the mechanisms underlying male-female differences in growth in invertebrates are less clear [3, 59]. We therefore used *Drosophila* larvae as a model to study the mechanisms underlying SSD. We identified clear male-female differences in the control of cell and body size that precede the differentiation of adult sexual morphology (*eg*. sex combs, abdominal pigmentation, genitalia). Since the duration of larval growth does not significantly differ between male and female larvae [5], these results implicate the sex-specific regulation of growth as a key determinant of SSD in *Drosophila*. While previous studies showed that master sex determination gene *Sxl* contributes to sex differences in body size [23], it was unclear whether these effects on growth were mediated by Sxl’s regulation of the sex determination pathway, or the process of dosage compensation. Also, Sxl’s role as a master sex determination gene is not conserved in all insects [60], suggesting other genes may contribute to SSD in these other species.

In our study, we show that sex determination gene *tra* contributes to SSD in *Drosophila*. This suggests that the sex-specific regulation of growth is at least partly independent of dosage compensation, as Tra does not regulate this process [30]. Since *tra*’s role in sex determination is widely conserved in insects, many of which show SSD, the sex-specific regulation of growth by Tra may be a conserved mechanism to create dimorphic body size across many insect species [61, 62]. It is important to note, however, that in spite of our results demonstrating an important role for Tra in creating SSD in *Drosophila*, loss of Tra function in females does not fully ‘masculinize’ body size, as *tra* mutant females remain significantly larger than wild-type males. There must therefore be other genes that contribute to increased female body size. One obvious candidate is *Sxl*, where *Sxl* mutant females have a male-like body size. The *tra*-independent effects of sex on body size may therefore be regulated by Sxl. This could occur in one of two ways: first, by Sxl acting on targets in addition to Tra, or second, by the effects of Sxl on dosage compensation. A recent study by Evans and Cline [63] showed that one female-specific behaviour, ovulation, was controlled by Sxl in a *tra*-independent manner. This ‘*tra*-insufficient feminization’ branch of the pathway does not cause any misregulation of the dosage compensation pathway, providing strong evidence that additional, as yet unknown, targets of Sxl mediate its effects on ovulation. In the case of SSD, then, other targets of Sxl may explain the *tra*-independent effects of sex on size. In addition to potential targets other than *tra*, the effects of Sxl on SSD may alternatively be mediated by its regulation of dosage compensation. In females, the presence of Sxl prevents the activation of the dosage compensation complex, whereas absence of Sxl in males allows dosage compensation to be activated to promote male development [11]. Loss of Sxl in females causes the inappropriate activation of this complex. Thus the decreased body size of *Sxl* mutant females may be explained by the ectopic activation of the dosage compensation complex. In the future, it will be interesting to dissect the individual contributions of *Sxl*, *tra*, the dosage compensation complex, and additional *Sxl* targets, to the control of male-female differences in body size in *Drosophila*. Further, it will be interesting, where possible, to determine whether these genes perform similar roles in SSD in other insect species.

One key finding from our work is that sex differences in body growth are regulated by Tra independently of sexual differentiation, behaviour and reproduction. To date, most studies have shown that Tra’s effects on sexual development are mediated by its known targets *dsx* and *fru* [43]. Together, *dsx* and *fru* regulate most aspects of sexual development and behaviour. However, our data shows that Tra’s effects on body size are independent of *dsx* and *fru*. Combined with our data showing that masculinizing or feminizing the gonads or germline has no effect on body size, this shows that that sex differences in body size are not simply a consequence of sexual differentiation, reproduction and behaviour. Instead, SSD in *Drosophila* is regulated by Tra in a separate pathway, separate from other sex determination genes and aspects of sexual dimorphism. One possible explanation for SSD to be regulated separately of other aspects of sexual development is that while increased female body size is an important sexual trait, as it is related to fecundity [64], the inability to adjust body size in response to environmental factors such as low nutrition can compromise survival during larval life [47]. Therefore, unlike aspects of sexual dimorphism that must be fixed to permit reproduction (*eg*. gonad and germline differentiation, female neural circuits for egg-laying), body size must show a higher degree of plasticity. Indeed, studies have shown that male genital discs in *Drosophila* are less sensitive to growth perturbation than other imaginal discs [65]. We therefore propose that sexually dimorphic body growth is regulated independently from other aspects of sexual differentiation to allow body size to be co-ordinated with environmental conditions.

Another finding from our work is that Tra function in the fat body can regulate the growth of other tissues to influence body size in a non cell-autonomous manner. Previous studies have also identified non cell-autonomous interactions that determine the sex of the genital disc, the development of the male-specific muscle of Lawrence, or sexual dimorphism in the gonad [66–69]. Combined with our data that sex differences in body size are also regulated in a non cell-autonomous manner, this suggests that in *Drosophila*, like in mammals, some aspects of sex determination and sexual dimorphism are regulated in a non cell-autonomous manner. Our identification of sex differences in the secretion of dILP2 suggest that this conservation extends to the cell-cell signaling pathways that mediate growth, as sex hormones in mammals are known to control male-female differences in body size via regulation of the growth hormone (GH)/insulin-like growth factor 1 (IGF1) axis [59]. Several recent studies have shown that higher levels of circulating dILPs can increase body growth by augmenting IIS activity [70,71]. Our findings therefore suggest a model of sex-specific growth in *Drosophila* in which the sex of the fat body, as determined by the presence (females) or absence (males) of Tra, is one contribution to the sex differences in body size via regulation of dILP secretion. Higher dILP secretion in females leads to elevated IIS activity, and consequently an increase in body size. This model of increased female body size is supported by data that flies lacking all three IPC-derived dILPs (*dilp2-3*,*5* triple mutants) show a 40% reduction in body weight in females, but no effect on body weight in males [72]. Similarly, female body size is more strongly affected than in males in animals with loss-of-function mutations in components of IIS such as *chico* or *InR* [73,74]. Together, these findings highlight the importance of sex as a critical determinant of dILP secretion, and IIS-mediated body growth.

During larval development, the fat body responds to a variety of extrinsic and intrinsic cues such as nutrients and hormones to control body growth. For example, in response to nutrient input, the fat body releases an as-yet-unidentified factor into the larval hemolymph [33]. This secreted factor acts in an endocrine manner to control the release of dILPs from the IPC in the brain. Our studies have identified sex as an additional factor that alters the function of the fat body to influence body growth in a non cell-autonomous manner. In particular, we identified a role for the function of sex determination gene Tra in the fat body as one factor influencing SSD in flies. Yet it is unclear how Tra function in the fat body influences the molecular and physiological properties of this tissue to influence body size. Tra is a member of the conserved family of SR proteins. These proteins play well-characterized roles in the regulation of alternative splicing, and have also been shown to influence other aspects of RNA metabolism, such as regulation of mRNA translation [75–77]. Tra may therefore act in two ways in the fat body to control dILP2 release: 1) via sex-specific splicing to facilitate production or secretion of the secreted factor(s), or 2) in a more general mechanism by influencing mRNA translation to elevate production or secretion of these fat-to-brain signals. Although the regulation of dILP secretion is an established mechanism to regulate body growth, the molecules that are released by the fat body to control dILP release are only beginning to be identified. For example, the cytokine-like molecule *unpaired 2* (*upd2*), and the peptide hormone CCHa2 play roles in coupling fat body function to regulation of dILP secretion and body size [32, 78]. Similarly, Hedgehog was also identified as a factor that can control dILP secretion in an endocrine manner [31]. In adults, fat body-derived dILP6 or *dawdle*, an Activin-like ligand in the TGF-β superfamily, could both influence the secretion of IPC-derived dILPs [79, 80]. In addition, several neuropeptides and neurotransmitters have also been shown to regulate IPC activity and dILP release [81]. In the future, it will be interesting to determine whether Tra directly regulates any of these known secreted factors to control dILP2 release. However, an additional possibility is that *tra* does not directly regulate any secreted factors; instead, *tra*’s effects on growth may be mediated by effects on mRNA translation. Many studies have identified the regulation of mRNA translation in the fat body as a limiting factor for growth during development. For example, two studies identified significant effects of TOR and Myc in the fat body in promoting dILP release [33, 82]. TOR is an important regulator of mRNA translation, and Myc’s effects were thought to involve elevated levels of ribosome synthesis. A more recent study showed that stimulation of tRNA synthesis, and consequently mRNA translation, in the fat body could drive increased body growth [83]. Future studies will allow us to determine which of Tra’s molecular functions (splicing vs. mRNA translation) determine its contribution to fat body function and consequently growth. Given the increasing awareness of functional similarities between the fly fat body and mammalian liver/adipose tissue, our results suggest the intriguing possibility that the function of these important endocrine organs may be similarly regulated by sex to control systemic growth and physiology in mammals.

In addition to Tra’s non cell-autonomous effects on body size, we found that Tra also has cell- and organ-autonomous effects on size. While our data suggests that Tra’s effects on body size are independent of *fru* and *dsx*, since loss of neither gene affects overall body growth or non cell-autonomous growth, it is possible that Tra’s cell-autonomous effects on cell size are mediated by *dsx*. In the larval fat body, we identified a cell-autonomous requirement for *dsx* in both males and females to promote growth in fat body cells. We believe the reason that these cell-autonomous effects of *dsx* on cell size do not affect overall body size is due to the restricted nature of Dsx expression in larvae. Indeed, two studies showed that Dsx expression in larvae is limited to the fat body, CNS, gonads, some regions of the gut, and subsets of imaginal discs [37,39]. However, in spite of the lack of effect on overall body size, previous studies in *Drosophila* have identified a role for *dsx* in regulating organ size in other tissues. For example, expression of the male- or female-specific isoforms of Dsx (Dsx^M^ and Dsx^F^, respectively) control the sex-specific growth of the genital disc via Wingless and Decapentaplegic signaling [84]. In addition, Dsx^F^ has been shown to promote sex-specific programmed cell death in both the larval ventral nerve cord, and in male-specific gonadal precursor cells [85–87]. Our findings identify an additional mechanism by which Dsx controls organ size: regulation of cell growth. While the molecular mechanism by which Dsx controls cell size is unclear, Dsx has been shown to control horn size in stag beetles by regulating tissue sensitivity to a circulating hormone, juvenile hormone [88]. Interestingly, a recent paper identified the *insulin receptor* (*InR*) and the *ecdysone receptor* (*EcR*) as potential Dsx targets [89]. Since both pathways have been shown to control fat body cell size [47, 82], Dsx may influence fat body cell growth by regulating tissue sensitivity to circulating dILPs or the steroid hormone ecdysone, integrating signals from both the primary sex-determining signal (X:A) and circulating hormones to control tissue growth. In the future, it will be interesting to determine whether the integration of sex and environmental cues is a general feature of Dsx-mediated tissue growth, or whether this mechanism is limited to specific tissues, such as the fat body.

In conclusion, our studies identify Tra as one regulator of sex differences in growth and body size. Moreover, we provide the first link between Tra and IIS in the control of sex differences in body growth. Interestingly, sexual dimorphism in phenotypes such as stress resistance, immune responses and lifespan have been noted in *Drosophila* [90–95]. These phenotypes are also affected by altering IIS [96–99]. Tra may therefore control sexual dimorphism in a wide variety of phenotypes via regulation of dILP secretion and IIS activity. Deregulation of insulin secretion and IIS activity have been implicated in diseases such as diabetes and cancer [45, 100]. Interestingly, sex differences in incidence have been previously reported for both diabetes and some forms of cancer [101, 102]. Thus future studies on the link between sex and insulin secretion/IIS activity may explain why one sex is predisposed to these diseases.

## Materials and Methods

### Fly stocks

Larvae were raised on food at a density of 50 larvae per vial at 25°C [83, 103]. The following fly GAL4 stocks were used in this study: *da-GAL4*, *r4-GAL4* (fat body), *cg-GAL4* (fat body), *elav-GAL4* (neurons), *repo-GAL4* (glia), *P0206-GAL4* (ring gland), *Mef2-GAL4* (muscle), *Act5c-GAL4* (ubiquitous), *en-GAL4* (posterior compartment of the wing), *nos-GAL4* (germline), *c587-GAL4* (gonad). We used the following UAS lines: *UAS-tra2-RNAi* (TriP), *UAS-tra2-RNAi* (VDRC), *UAS-tra*, *UAS-dsx-RNAi* (TRiP). The following mutant strains were used: *w* ^*1118*^, *foxo* ^*Δ94*^, *tra* ^*1*^ */TM6B*, *Df(3L)st-j7/TM6B*, *dsx* ^*1*^ */TM6B*, *Df(3R)dsx* ^*15*^, *tud* ^*1*^ */CyO*::*GFP*, *fru* ^*F*^ */TM6B*, *fru* ^*4-40*^ */TM6B*, *fru* ^*Δtra*^ */TM6B*, *pten* ^*100*^ *;CyO*::*GFP;MKRS/TM6B*. We used the following stocks for *flp*-out experiments: *hsflp;;UAS-tra2-RNAi*, *hsflp;UAS-tra*, *hsflp;;UAS-dsx-RNAi*, *act>stop>CD2>stop>GAL4*. Larvae were sexed using gonad size. Where gonad size could not be used to sex larvae (*eg*. *dsx* or *tra* mutants, *da>UAS-tra*, *Mef2>UAS-tra*, *Act5c>tra2-RNAi* or *Act5c>UAS-tra*), males with a GFP on the X chromosome (*Ubiquitin-GFP*) were crossed to the virgin females of the correct genotype. In the progeny of the cross, females were GFP-positive and males were GFP-negative [5].

### Pupal volume

Pupal volume was measured as previously described [82]. n>60 per genotype.

### Cell size, compartment size and cell number

Measured as previously described [83, 103]. n>40 per genotype.

### Adult weight

Five-day-old adult flies lacking gonads were weighed in groups of six in 1.5 ml tubes on an analytical balance. The gonads were removed prior to weighing by dissection; n>30 per genotype.

### Feeding assays

96 hr larvae were fed for the indicated amounts of time on yeast paste containing 0.05% Bromophenol blue. After feeding for the desired amount of time, ten larvae were isolated in a 1.5 ml tube, with eight tubes per sex collected in total. 250 μl of PBS was added to the tube and the larvae were homogenized with a micropestle. The lysate was cleared by centrifugation at 5000 rpm for 1 min, then the absorbance at 595 nm was measured in a spectrophotometer.

### qRT-PCR

Total RNA was extracted from larval tissues, then DNase-treated and reverse transcribed using Superscript II, as previously described [83, 103].

### Western blotting

Whole larval extracts were prepared as previously described [83, 103]. The P-Akt and total Akt antibodies were obtained from Cell Signaling (#4054 and #9272).

### Anti-FOXO immunohistochemistry

Anti-FOXO antibody was applied to fat bodies dissected from larvae 110 hr AEL (25°C) at a dilution of 1:500, as previously described [80].

### Rapamycin feeding

Larvae were grown on rich food containing either DMSO or rapamycin, as previously described [83, 103].

### Statistics

All data were analyzed using R Studio using the code described below.

Student’s t-test:

a <- filename$genotype1

b <- filename$genotype2

t.test(a,b)

One-way ANOVA:

aov.PV <- aov(Pupal_Volume ~ Genotype, data = filename)

ls(aov.PV)

summary(aov.PV)

TukeyHSD(aov.PV)

Two-way ANOVA with interaction term:

int <- aov(Pupal_Volume ~ Sex + Treatment + Sex*Treatment, data = filename)

summary(int)

TukeyHSD(int)

## Supporting Information

S1 Fig Larval feeding behaviour does not differ between males and females. (A) Food intake was quantified by measuring the absorbance (595 nm) of a larval lysate after larvae were allowed to feed on yeast paste containing 0.05% bromophenol blue for the indicated amounts of time. No significant differences in food intake between the sexes were observed at any time point (p = 0.05, 0.48, 0.34, respectively; Student’s t-test). (B) Mouth hook contractions in 30 sec also did not differ between male and female larvae tested at 96 hr after egg laying (p = 0.146, n>20; Student’s t-test). (C) Global expression of the *UAS-tra2-RNAi* transgene using *Act5c-GAL4* transforms a female into a phenotypic male (*eg*. abdominal pigmentation, genitalia). (D) Body size is also significantly decreased in these *Act5c>tra2-RNAi* females (p = 0.028;0.009; one-way ANOVA followed by Tukey HSD post-hoc test), but not in males (p = 0.68;0.2, one-way ANOVA followed by Tukey HSD post-hoc test). * indicates a significant difference, N.S. means not different from both control genotypes. The p-values indicated are listed in the following order: difference between the GAL4/UAS genotype and the GAL4 control; difference between the GAL4/UAS genotype and the UAS control. A list of all p-values obtained from the Tukey HSD post-hoc test is provided in S1 Table.
(TIF)

S2 Fig *tra* expression also influences adult body weight. (A) Adult body weight in *tra* mutant females is significantly decreased compared to control females (p = 3.3 x 10^−6^; 4 x 10^−7^, one-way ANOVA followed by Tukey HSD post-hoc test). *tra* mutant male body size is not different from controls (p = 0.98; 1, one-way ANOVA followed by Tukey HSD post-hoc test). (B) Male adult body weight is significantly increased in animals with ubiquitous expression of *UAS-tra* (p = 0; 0, one-way ANOVA followed by Tukey HSD post-hoc test), while female body weight does not change (p = 0.97; 0.23, one-way ANOVA followed by Tukey HSD post-hoc test). Weights shown are for groups of six flies, all flies were weighed at five days old, and had their gonads removed by dissection prior to weighing. * indicates a significant difference, N.S. means not significantly different from both control genotypes. The p-values indicated are listed in the following order: difference between the first control genotype and the experimental control;difference between the second control genotype and the experimental control. A list of all p-values obtained from the Tukey HSD post-hoc test is provided in S1 Table.
(TIF)

S3 Fig Tissue-specific loss of *tra2* has no effect on body growth in males. (A) Expression of an independent *tra2-RNAi* line with *en-GAL4* also causes a 10% reduction in the posterior:anterior ratio in the adult wing in females (p = 1.4 x 10^−28^, Student’s t-test), but no effect in males (p = 0.3, Student’s t-test). (B) Overexpression of Tra in the posterior compartment of the wing does not increase the posterior:anterior ratio in the adult wing in females, and may even decrease it (p = 0.0023, Student’s t-test). Overexpression of Tra in males using *en-GAL4* has no effect on posterior:anterior ratio (p = 0.304, Student’s t-test). (C) Using *r4-GAL4* to express an independent *tra2-RNAi* line reduces body size in females (p = 0;0, one-way ANOVA followed by Tukey HSD post-hoc test), but does not affect body growth in males (p = 0.87;0.53, one-way ANOVA followed by Tukey HSD post-hoc test). * indicates a significant difference, N.S. means not significantly different from both control genotypes. The p-values indicated are listed in the following order: difference between the GAL4/UAS genotype and the GAL4 control;difference between the GAL4/UAS genotype and the UAS control. A list of all p-values obtained from the Tukey HSD post-hoc test is provided in S1 Table.
(TIF)

S4 Fig Tissue-specific expression of *tra2-RNAi* does not affect body size in males. (A) Expression of *tra2-RNAi* in neurons, glia, ring gland (RGl), and muscle had no effect on body size in females (p = 0.25;0.44 (elav), 0.87;0.95 (repo), 0.99;0.99 (P0206) and 0.35;1.4 x 10^−4^ (Mef2) respectively, one-way ANOVA followed by Tukey HSD post-hoc test). (B) Expressing *tra2-RNAi* using different tissue-specific GAL4 drivers does not affect male body size (p = 0.13;0.43 (r4), 0.99;0.93 (cg), 0.0008;0.99 (elav), 0.01;0.24 (repo), 0.63;0.2 (P0206), 0.4;0.96 (Mef2), respectively, one-way ANOVA followed by Tukey HSD post-hoc test). N.S. means not significantly different from both control genotypes. The p-values indicated are listed in the following order: difference between the GAL4/UAS genotype and the GAL4 control, then difference between the GAL4/UAS genotype and the UAS control. A list of all p-values obtained from the Tukey HSD post-hoc test is provided in S1 Table.
(TIF)

S5 Fig Tissue-specific overexpression of Tra does not augment body size in wild-type males and females. (A) In females, ubiquitous expression of Tra using *Act5c-GAL4* increases body size (p = 4 x 10^−5^;0.015; one-way ANOVA followed by Tukey HSD post-hoc test). Tissue-specific expression of Tra in fat body, neurons, ring gland, or muscle does not similarly increase body size (p = 1;0.07 (r4), 0.79;0.82 (cg), 0.94;1 (elav), 0.002;0.97 (P0206) and 0.99;0.007 (Mef2); one-way ANOVA followed by Tukey HSD post-hoc test). (B) Although ubiquitous expression of Tra in males increases body size in males (p = 0.01;0.003; one-way ANOVA followed by Tukey HSD post-hoc test), tissue-specific Tra expression does not (p = 0.76;0.98 (r4), 0.8;0.99 (cg), 0.85;0.99 (elav), 0.99;0.99 (P0206) and 0.99;0.28 (Mef2); one-way ANOVA followed by Tukey HSD post-hoc test). * indicates a significant difference, N.S. means not significantly different from both control genotypes. The p-values indicated are listed in the following order: difference between the GAL4/UAS genotype and the GAL4 control, then difference between the GAL4/UAS genotype and the UAS control. A list of all p-values obtained from the Tukey HSD post-hoc test is provided in S1 Table.
(TIF)

S6 Fig *tra’s* body growth effects are not mediated by the germline or gonad. (A,B) Gonad- (*c587-GAL4*) or germline-specific (*nos-GAL4*) expression of the *UAS-tra2-RNAi* transgene does not affect body size in females (p = 0.99;0.008, 0.98;0.06; one-way ANOVA followed by Tukey HSD post-hoc test) or in males (p = 0.9;0.055, 1;0.37; one-way ANOVA followed by Tukey HSD post-hoc test), respectively. (C) Pupal volume was measured in the progeny of *w* ^*1118*^ males crossed to either *tud* ^*1*^ */tud* ^*1*^ females or *tud* ^*1*^ */+* females. Progeny of the *tud* ^*1*^ */tud* ^*1*^ homozygous mothers will lack a germline, whereas progeny of the *tud* ^*1*^ */+* mothers will have a germline. No significant decrease in pupal volume was observed in females or males lacking a germline compared to animals with a germline (p = 0.31 and 0.41, respectively; one-way ANOVA followed by Tukey HSD post-hoc test). N.S. means not significantly different from both control genotypes. The p-values indicated are listed in the following order: difference between the GAL4/UAS genotype and the GAL4 control (or first control genotype), then difference between the GAL4/UAS genotype and the UAS control (or second control genotype). A list of all p-values obtained from the Tukey HSD post-hoc test is provided in S1 Table.
(TIF)

S7 Fig Male-female differences in systemic insulin signaling and *dilp* expression. (A) Male and female larvae grown in food with rapamycin, a specific Target-of-Rapamycin (TOR) pathway inhibitor, had a 25% SSD, similar to the 26% SSD of males and females raised without rapamycin (p = 0; 0, two-way ANOVA with Tukey HSD post-hoc test). (B) Levels of phospho-Akt (P-Akt) were quantified in male and female larvae analyzed 96 hr or 120 hr after egg laying at 25°C (AEL). (C) Females have significantly higher levels of P-Akt at 120 hr AEL (p = 0.005, Student’s t-test). (D,D’) Anti-FOXO antibody was applied to female, and male fat bodies dissected from larvae collected 110 hr AEL. (D) In females, anti-FOXO localization is distributed evenly between the cytoplasm and nucleus, whereas in males (D’) the localization is predominantly nuclear. (E) Quantification of FOXO localization from (D) showing that males have a significantly higher nuclear:cytoplasmic ratio of FOXO compared to females (p = 3.5 x 10^−43^, Student’s t-test). (F) Male and female transcript levels of *foxo* target genes *InR*, *4E-BP* and *dilp6* were analyzed in larval carcasses devoid of fat body in the indicated genotypes by qRT-PCR at 120 hr AEL. Levels of *InR*, *4E-BP* and *dilp6* were not different between controls and females expressing the *tra2-RNAi* transgene in the fat body (p = 0.06;0.09, 0.08;0.14 and 0.08;0.04, respectively, Student’s t-test). Levels of all three genes were similarly unchanged in males (p = 0.002;0.36, 0.11;0.12 and 0.17;0.25, respectively; Student’s t-test). (G) Fat body-specific expression of *tra2-RNAi* does not significantly affect *dilp2*, *dilp3* or *dilp5* transcript levels in carcasses devoid of fat body in the indicated genotypes in either females or in males at 120 hr AEL (Fem: p = 0.081;0.004, 0.088;0.01 and 0.07;0.007. Male: p = 0.06;0.02, 0.28;0.33, 0.004;0.007, Student’s t-test). * indicates a significant difference, N.S. means not significantly different from both control genotypes. The p-values indicated are listed in the following order: difference between the GAL4/UAS genotype and the GAL4 control, then difference between the GAL4/UAS genotype and the UAS control. A list of all p-values obtained from the Tukey HSD post-hoc test is provided in S1 Table.
(TIF)

S1 Table A list of all p-values from one- and two-way ANOVA analysis. All F-values from one- and two-way ANOVA analysis are shown. Also included are the p-values from the pairwise comparisons generated by the Tukey HSD post-hoc test.
(XLSX)
