# Supplementary figures and images for: The Sex Determination Gene transformer Regulates Male-Female Differences in Drosophila Body Size
Source: PLoS Genet. 2015 Dec 28;11(12):e1005683. doi: 10.1371/journal.pgen.1005683 (PMC4692505; doi:10.1371/journal.pgen.1005683)

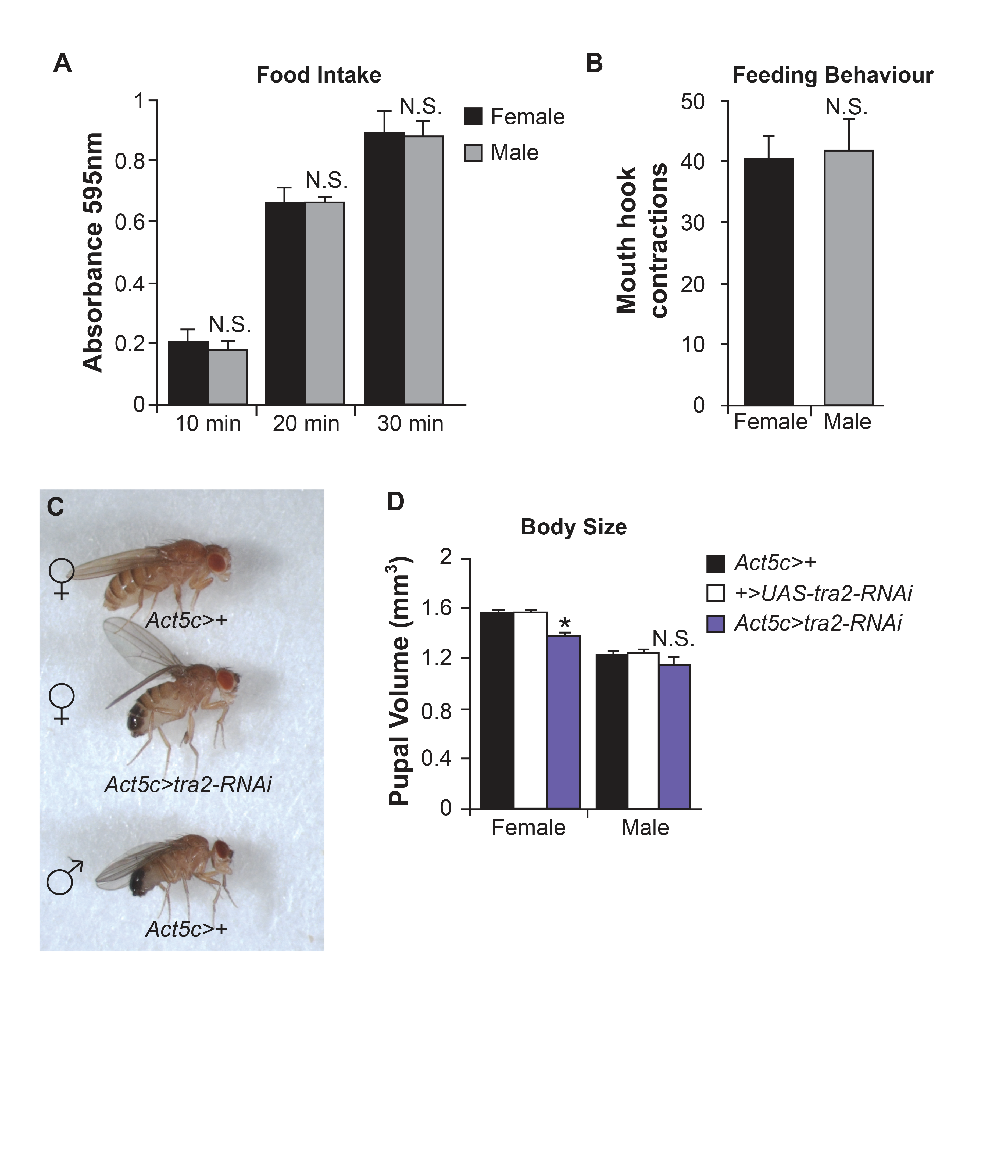

Supplement: S1 Fig — (A) Food intake was quantified by measuring the absorbance (595 nm) of a larval lysate after larvae were allowed to feed on yeast paste containing 0.05% bromophenol blue for the indicated amounts of time. No significant differences in food intake between the sexes were observed at any time point (p = 0.05, 0.48, 0.34, respectively; Student’s t-test). (B) Mouth hook contractions in 30 sec also did not differ between male and female larvae tested at 96 hr after egg laying (p = 0.146, n>20; Student’s t-test). (C) Global expression of the UAS-tra2-RNAi transgene using Act5c-GAL4 transforms a female into a phenotypic male (eg. abdominal pigmentation, genitalia). (D) Body size is also significantly decreased in these Act5c>tra2-RNAi females (p = 0.028;0.009; one-way ANOVA followed by Tukey HSD post-hoc test), but not in males (p = 0.68;0.2, one-way ANOVA followed by Tukey HSD post-hoc test). * indicates a significant difference, N.S. means not different from both control genotypes. The p-values indicated are listed in the following order: difference between the GAL4/UAS genotype and the GAL4 control; difference between the GAL4/UAS genotype and the UAS control. A list of all p-values obtained from the Tukey HSD post-hoc test is provided in S1 Table. (TIF) [file pgen.1005683.s001.tif]

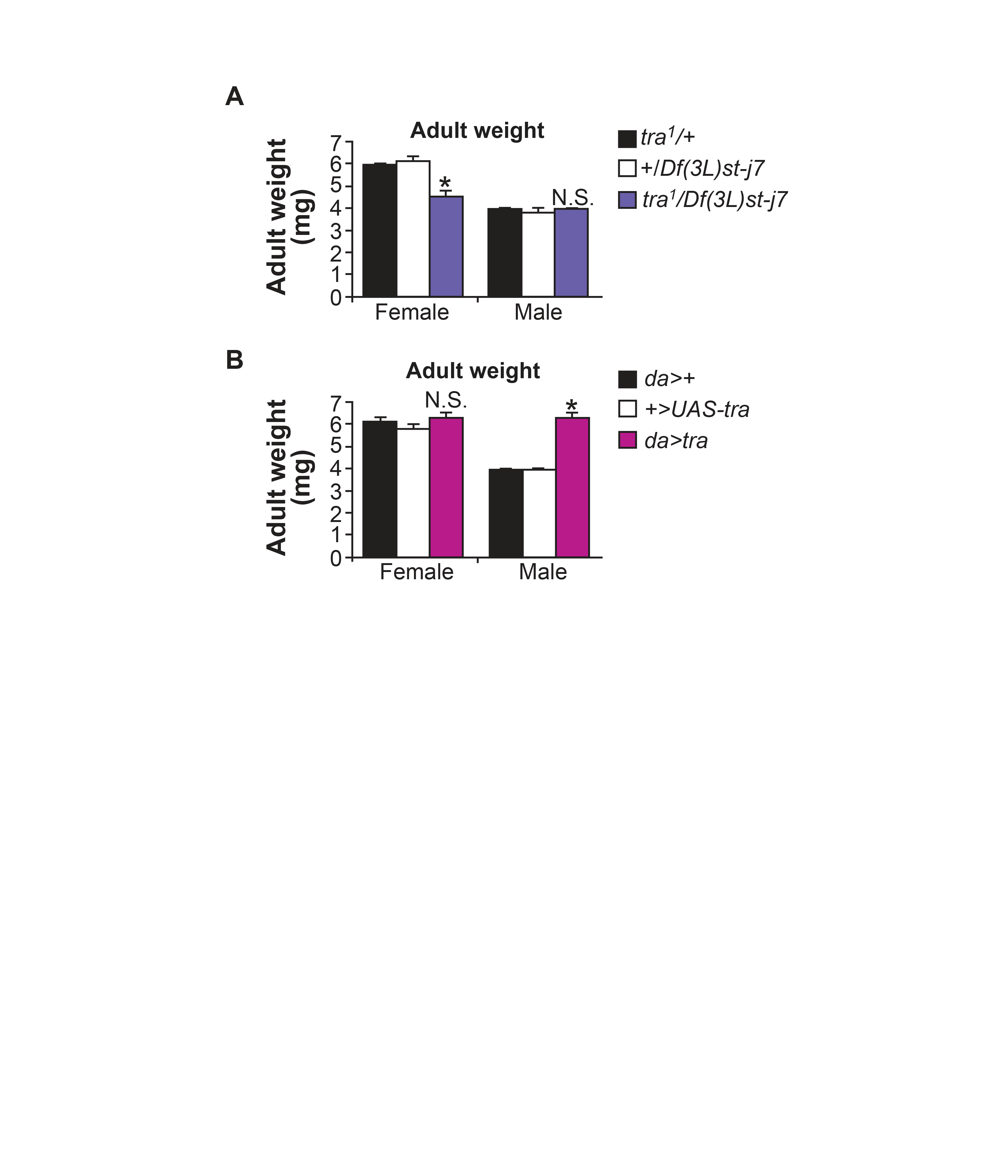

Supplement: S2 Fig — (A) Adult body weight in tra mutant females is significantly decreased compared to control females (p = 3.3 x 10^−6^; 4 x 10^−7^, one-way ANOVA followed by Tukey HSD post-hoc test). tra mutant male body size is not different from controls (p = 0.98; 1, one-way ANOVA followed by Tukey HSD post-hoc test). (B) Male adult body weight is significantly increased in animals with ubiquitous expression of UAS-tra (p = 0; 0, one-way ANOVA followed by Tukey HSD post-hoc test), while female body weight does not change (p = 0.97; 0.23, one-way ANOVA followed by Tukey HSD post-hoc test). Weights shown are for groups of six flies, all flies were weighed at five days old, and had their gonads removed by dissection prior to weighing. * indicates a significant difference, N.S. means not significantly different from both control genotypes. The p-values indicated are listed in the following order: difference between the first control genotype and the experimental control;difference between the second control genotype and the experimental control. A list of all p-values obtained from the Tukey HSD post-hoc test is provided in S1 Table. (TIF) [file pgen.1005683.s002.tif]

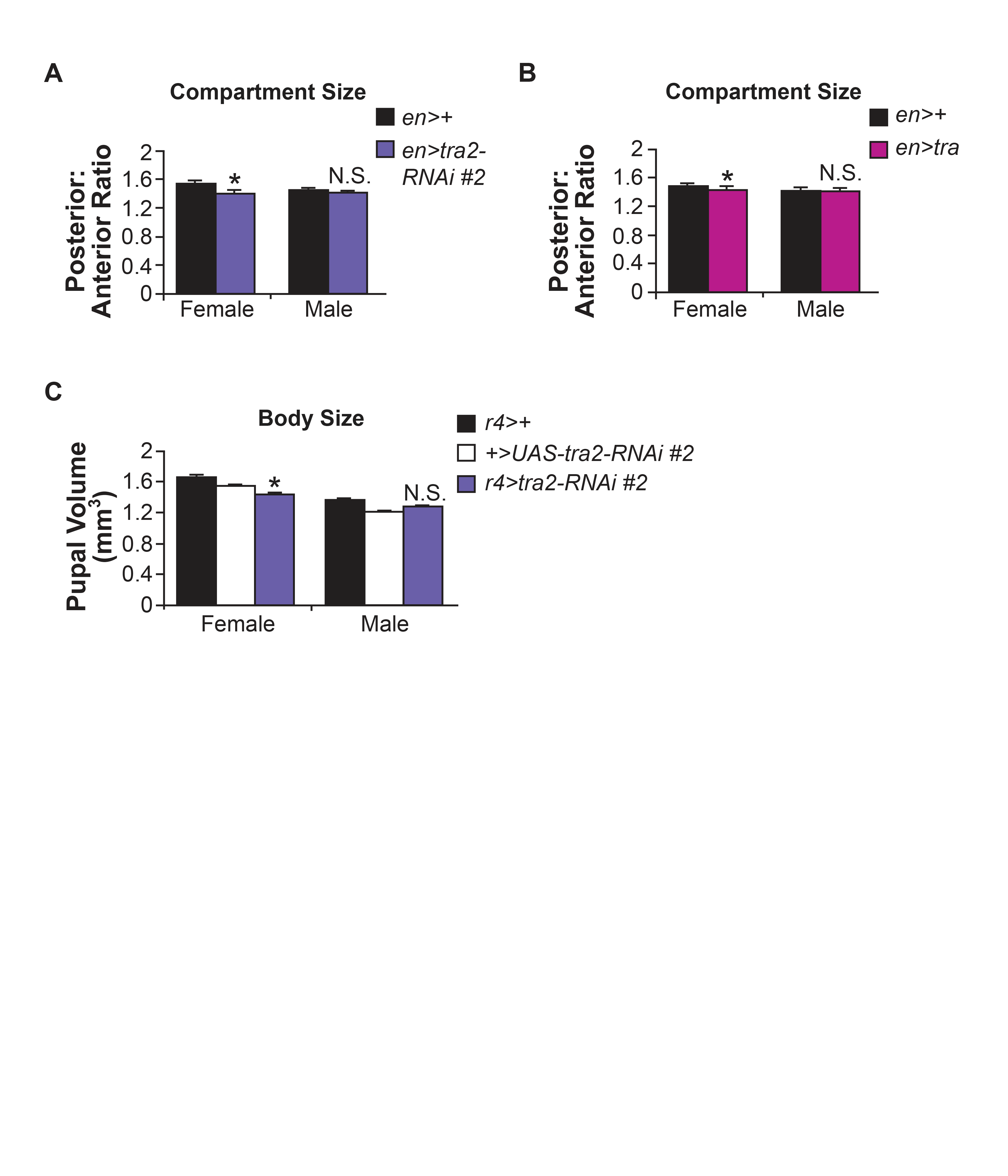

Supplement: S3 Fig — (A) Expression of an independent tra2-RNAi line with en-GAL4 also causes a 10% reduction in the posterior:anterior ratio in the adult wing in females (p = 1.4 x 10^−28^, Student’s t-test), but no effect in males (p = 0.3, Student’s t-test). (B) Overexpression of Tra in the posterior compartment of the wing does not increase the posterior:anterior ratio in the adult wing in females, and may even decrease it (p = 0.0023, Student’s t-test). Overexpression of Tra in males using en-GAL4 has no effect on posterior:anterior ratio (p = 0.304, Student’s t-test). (C) Using r4-GAL4 to express an independent tra2-RNAi line reduces body size in females (p = 0;0, one-way ANOVA followed by Tukey HSD post-hoc test), but does not affect body growth in males (p = 0.87;0.53, one-way ANOVA followed by Tukey HSD post-hoc test). * indicates a significant difference, N.S. means not significantly different from both control genotypes. The p-values indicated are listed in the following order: difference between the GAL4/UAS genotype and the GAL4 control;difference between the GAL4/UAS genotype and the UAS control. A list of all p-values obtained from the Tukey HSD post-hoc test is provided in S1 Table. (TIF) [file pgen.1005683.s003.tif]

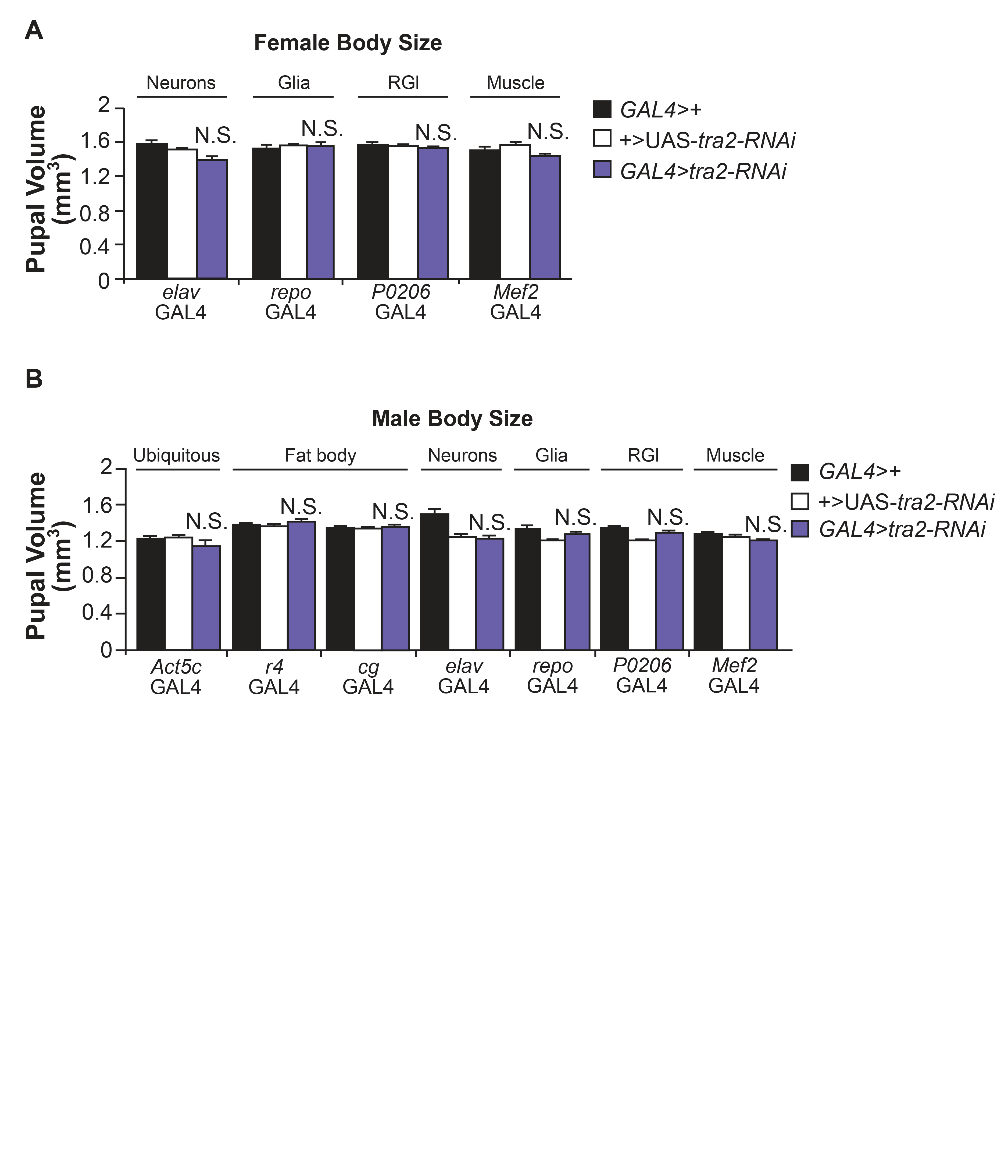

Supplement: S4 Fig — (A) Expression of tra2-RNAi in neurons, glia, ring gland (RGl), and muscle had no effect on body size in females (p = 0.25;0.44 (elav), 0.87;0.95 (repo), 0.99;0.99 (P0206) and 0.35;1.4 x 10^−4^ (Mef2) respectively, one-way ANOVA followed by Tukey HSD post-hoc test). (B) Expressing tra2-RNAi using different tissue-specific GAL4 drivers does not affect male body size (p = 0.13;0.43 (r4), 0.99;0.93 (cg), 0.0008;0.99 (elav), 0.01;0.24 (repo), 0.63;0.2 (P0206), 0.4;0.96 (Mef2), respectively, one-way ANOVA followed by Tukey HSD post-hoc test). N.S. means not significantly different from both control genotypes. The p-values indicated are listed in the following order: difference between the GAL4/UAS genotype and the GAL4 control, then difference between the GAL4/UAS genotype and the UAS control. A list of all p-values obtained from the Tukey HSD post-hoc test is provided in S1 Table. (TIF) [file pgen.1005683.s004.tif]

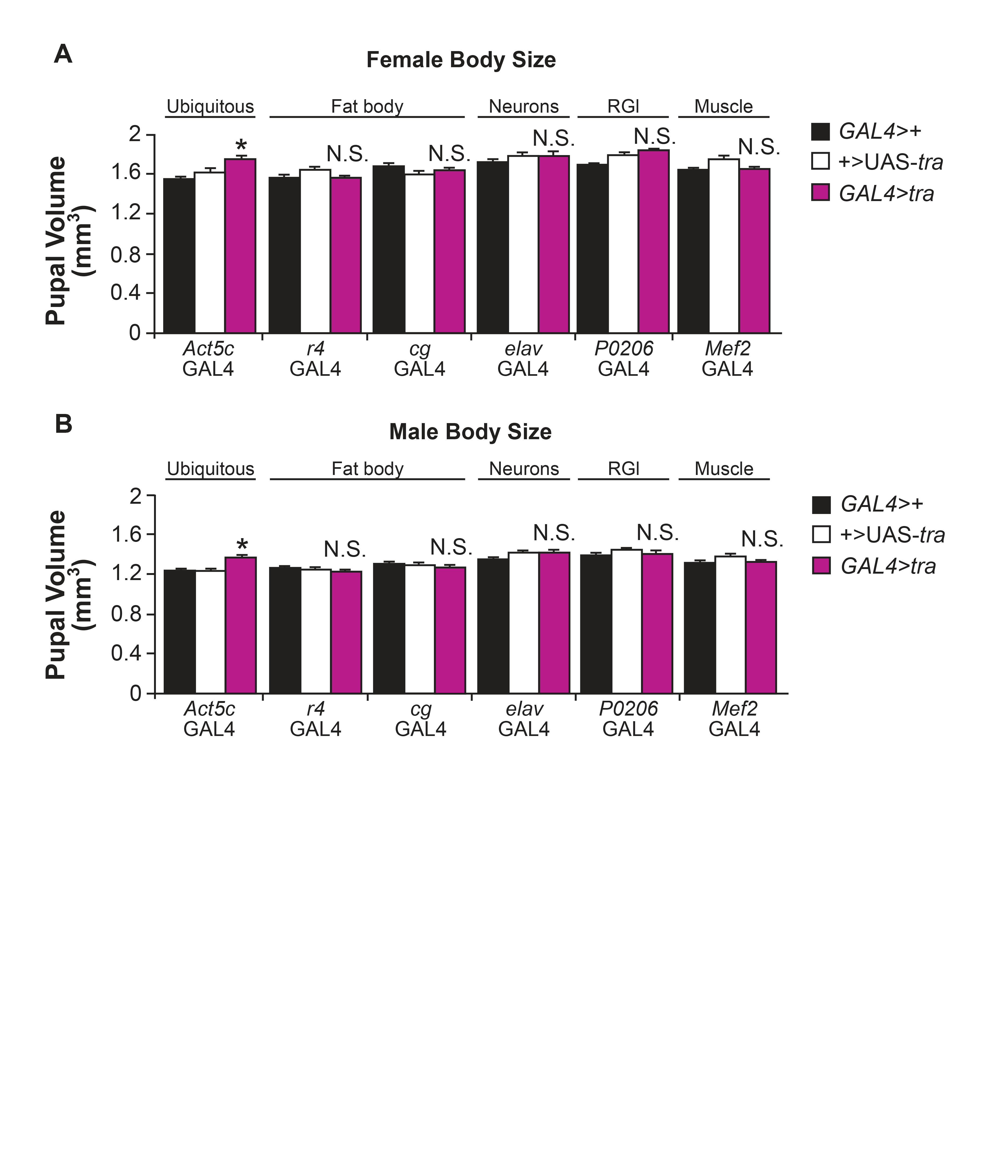

Supplement: S5 Fig — (A) In females, ubiquitous expression of Tra using Act5c-GAL4 increases body size (p = 4 x 10^−5^;0.015; one-way ANOVA followed by Tukey HSD post-hoc test). Tissue-specific expression of Tra in fat body, neurons, ring gland, or muscle does not similarly increase body size (p = 1;0.07 (r4), 0.79;0.82 (cg), 0.94;1 (elav), 0.002;0.97 (P0206) and 0.99;0.007 (Mef2); one-way ANOVA followed by Tukey HSD post-hoc test). (B) Although ubiquitous expression of Tra in males increases body size in males (p = 0.01;0.003; one-way ANOVA followed by Tukey HSD post-hoc test), tissue-specific Tra expression does not (p = 0.76;0.98 (r4), 0.8;0.99 (cg), 0.85;0.99 (elav), 0.99;0.99 (P0206) and 0.99;0.28 (Mef2); one-way ANOVA followed by Tukey HSD post-hoc test). * indicates a significant difference, N.S. means not significantly different from both control genotypes. The p-values indicated are listed in the following order: difference between the GAL4/UAS genotype and the GAL4 control, then difference between the GAL4/UAS genotype and the UAS control. A list of all p-values obtained from the Tukey HSD post-hoc test is provided in S1 Table. (TIF) [file pgen.1005683.s005.tif]

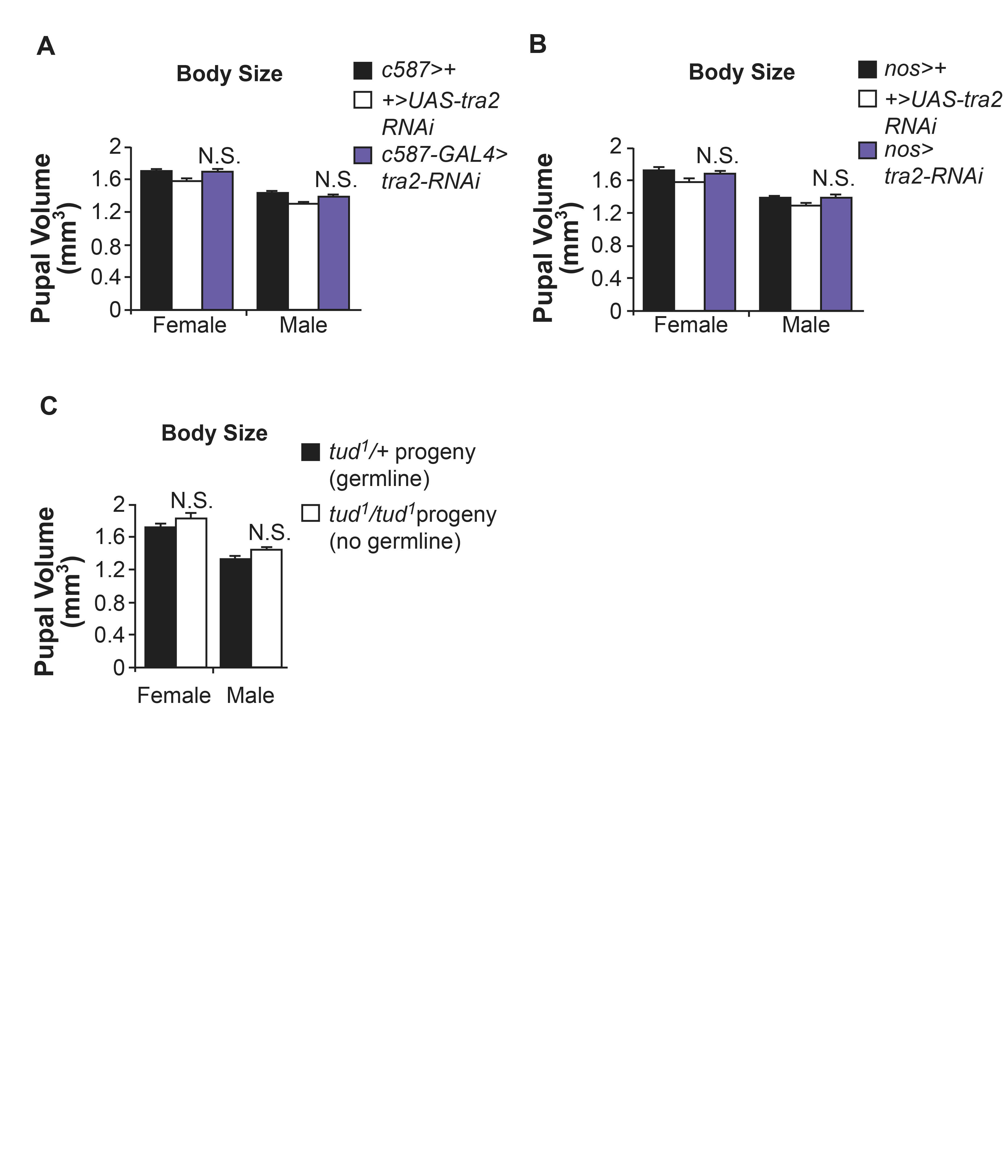

Supplement: S6 Fig — (A,B) Gonad- (c587-GAL4) or germline-specific (nos-GAL4) expression of the UAS-tra2-RNAi transgene does not affect body size in females (p = 0.99;0.008, 0.98;0.06; one-way ANOVA followed by Tukey HSD post-hoc test) or in males (p = 0.9;0.055, 1;0.37; one-way ANOVA followed by Tukey HSD post-hoc test), respectively. (C) Pupal volume was measured in the progeny of w 1118 males crossed to either tud 1 /tud 1 females or tud 1 /+ females. Progeny of the tud 1 /tud 1 homozygous mothers will lack a germline, whereas progeny of the tud 1 /+ mothers will have a germline. No significant decrease in pupal volume was observed in females or males lacking a germline compared to animals with a germline (p = 0.31 and 0.41, respectively; one-way ANOVA followed by Tukey HSD post-hoc test). N.S. means not significantly different from both control genotypes. The p-values indicated are listed in the following order: difference between the GAL4/UAS genotype and the GAL4 control (or first control genotype), then difference between the GAL4/UAS genotype and the UAS control (or second control genotype). A list of all p-values obtained from the Tukey HSD post-hoc test is provided in S1 Table. (TIF) [file pgen.1005683.s006.tif]

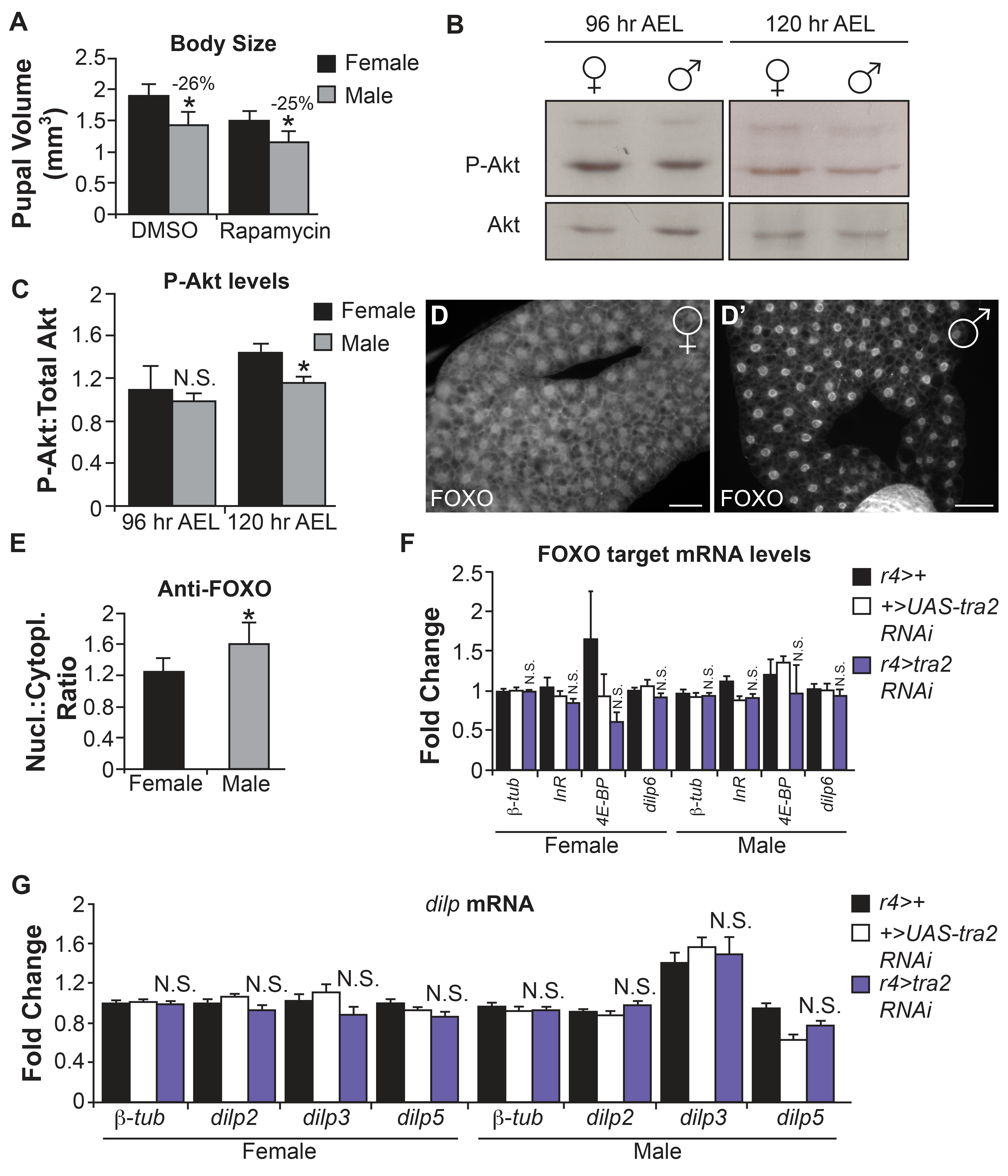

Supplement: S7 Fig — (A) Male and female larvae grown in food with rapamycin, a specific Target-of-Rapamycin (TOR) pathway inhibitor, had a 25% SSD, similar to the 26% SSD of males and females raised without rapamycin (p = 0; 0, two-way ANOVA with Tukey HSD post-hoc test). (B) Levels of phospho-Akt (P-Akt) were quantified in male and female larvae analyzed 96 hr or 120 hr after egg laying at 25°C (AEL). (C) Females have significantly higher levels of P-Akt at 120 hr AEL (p = 0.005, Student’s t-test). (D,D’) Anti-FOXO antibody was applied to female, and male fat bodies dissected from larvae collected 110 hr AEL. (D) In females, anti-FOXO localization is distributed evenly between the cytoplasm and nucleus, whereas in males (D’) the localization is predominantly nuclear. (E) Quantification of FOXO localization from (D) showing that males have a significantly higher nuclear:cytoplasmic ratio of FOXO compared to females (p = 3.5 x 10^−43^, Student’s t-test). (F) Male and female transcript levels of foxo target genes InR, 4E-BP and dilp6 were analyzed in larval carcasses devoid of fat body in the indicated genotypes by qRT-PCR at 120 hr AEL. Levels of InR, 4E-BP and dilp6 were not different between controls and females expressing the tra2-RNAi transgene in the fat body (p = 0.06;0.09, 0.08;0.14 and 0.08;0.04, respectively, Student’s t-test). Levels of all three genes were similarly unchanged in males (p = 0.002;0.36, 0.11;0.12 and 0.17;0.25, respectively; Student’s t-test). (G) Fat body-specific expression of tra2-RNAi does not significantly affect dilp2, dilp3 or dilp5 transcript levels in carcasses devoid of fat body in the indicated genotypes in either females or in males at 120 hr AEL (Fem: p = 0.081;0.004, 0.088;0.01 and 0.07;0.007. Male: p = 0.06;0.02, 0.28;0.33, 0.004;0.007, Student’s t-test). * indicates a significant difference, N.S. means not significantly different from both control genotypes. The p-values indicated are listed in the following order: difference between the GAL4/UAS [file pgen.1005683.s007.tif]
